# Supplementary material for: Assessment of the Genetic Structure and Diversity of Soybean (Glycine max L.) Germplasm Using Diversity Array Technology and Single Nucleotide Polymorphism Markers
Source: Plants (Basel). 2021 Dec 26;11(1):68. doi: 10.3390/plants11010068 (PMC8747349; doi:10.3390/plants11010068)
Supplement: Supplementary file 1 [file plants-11-00068-s001.zip › Supp. Figures and Tables.pdf]

Table S1: Quality parameter of the DArT-SNP markers

Parameter	SNP				DArT		
	Minimum	Maximum	Average		Minimum	Maximum	Average
MAF	0.05	0.50	0.14		0.05	0.50	0.30
Reproducibility	0.95	1.00	0.99		0.95	1.00	0.94
Call rate	0.89	1.00	0.97		0.89	1.00	0.99

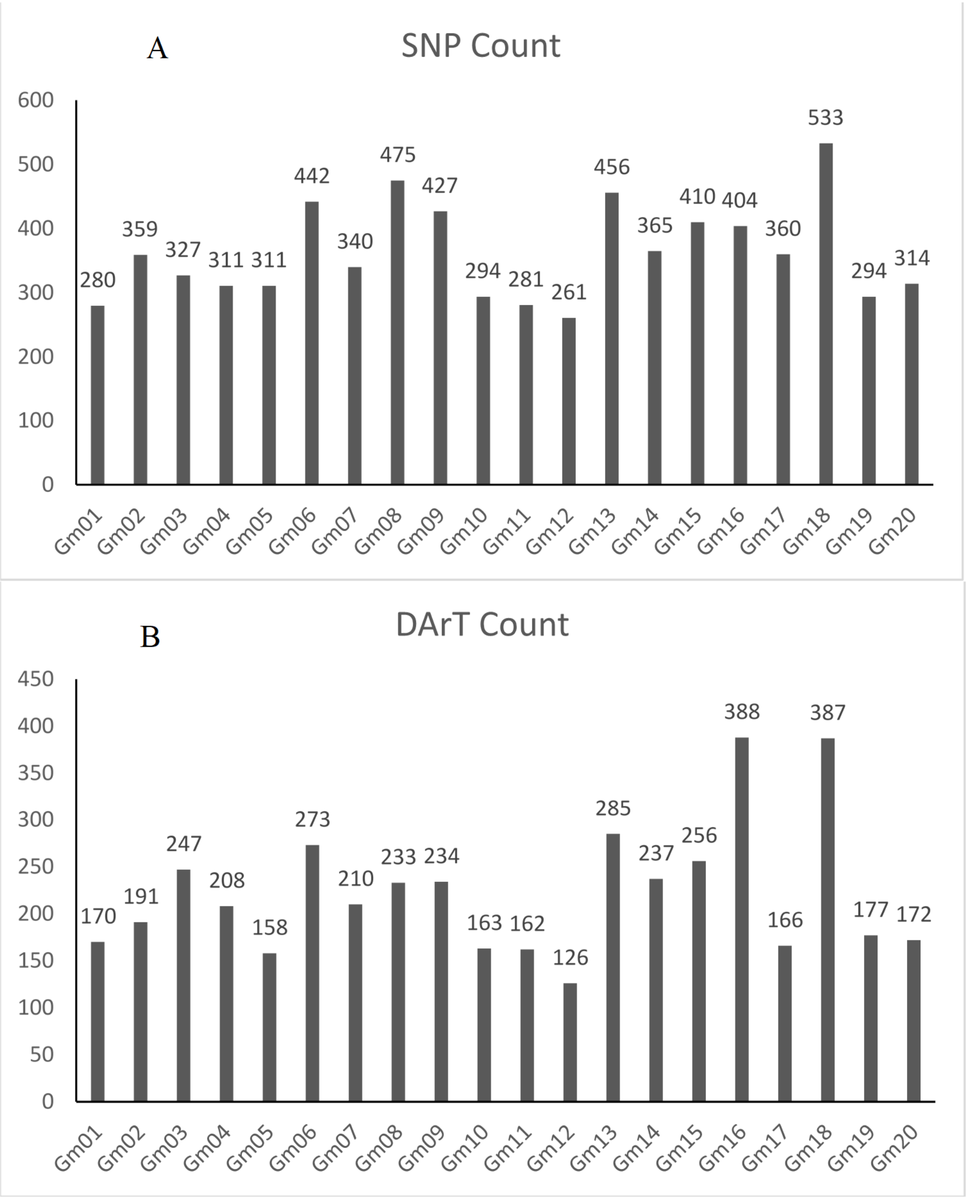

Figure S1. Distribution of SNPs across the 20 chromosomes of soybean. (a) SNP markers and (b) DArT markers.

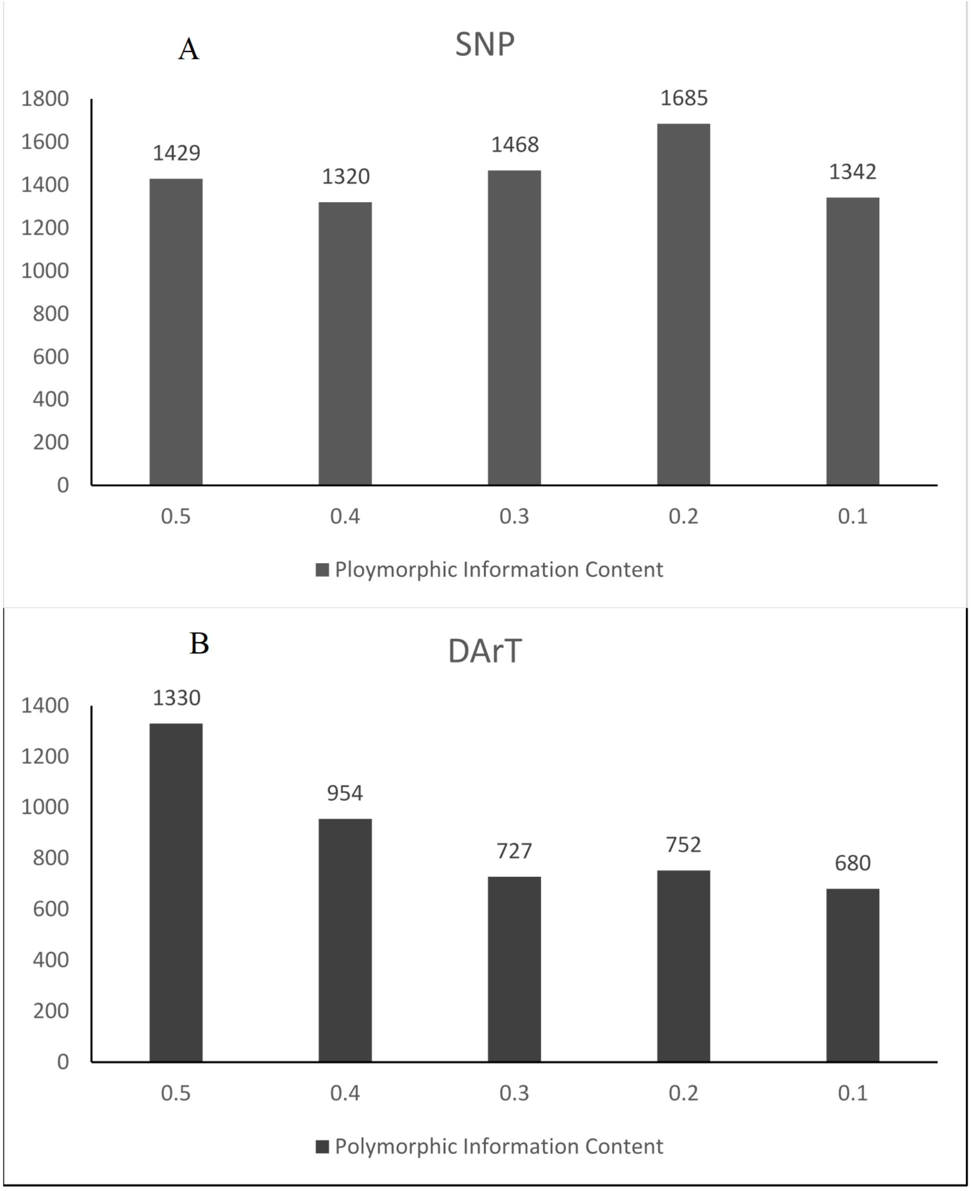

Figure S2. The polymorphic information content of markers. (a) SNP markers and (b) DArT markers.

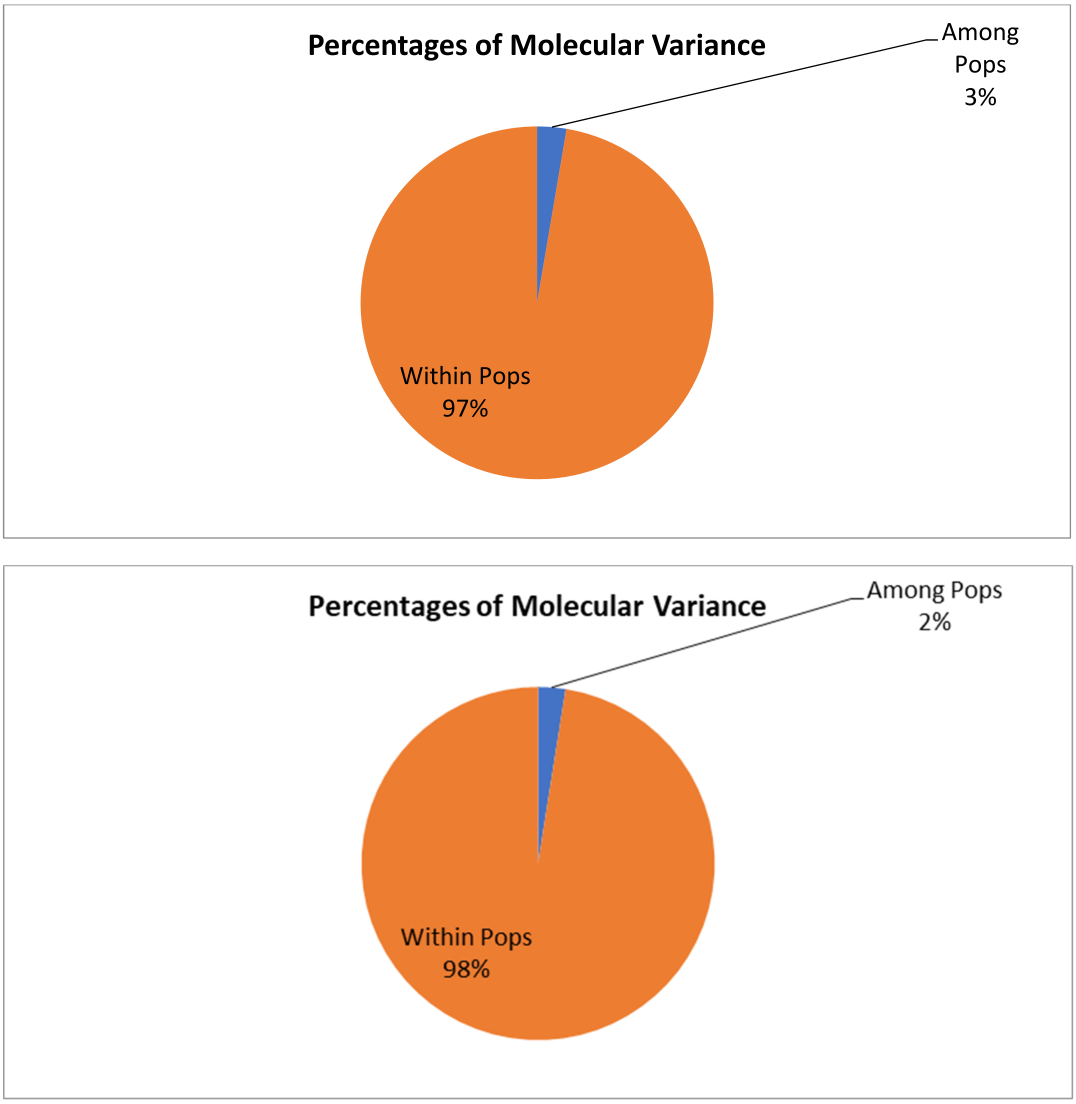

Figure S3: Percentages of molecular variance within and among populations. (a) SNP markers (b) DArT markers

Table S2: Allelic patterns across the populations for SNP and DArT markers

Population	Number of alleles (Na)	Number of effective alleles (Ne)	Shannon's diversity index (I)	Diversity (h)	Unbiased diversity (uh)
	**SNP**				
1	0.93	1.33	0.26	0.18	0.27
2	1.30	1.45	0.38	0.26	0.35
3	1.05	1.35	0.28	0.19	0.29
4	0.63	1.19	0.13	0.09	0.19
5	1.98	1.59	0.52	0.34	0.35
6	1.89	1.54	0.48	0.32	0.33
7	0.73	1.21	0.15	0.11	0.21
8	1.86	1.54	0.48	0.32	0.33
9	0.84	1.32	0.22	0.16	0.32
10	1.93	1.56	0.49	0.33	0.34
11	1.29	1.44	0.36	0.25	0.33
12	1.29	1.44	0.36	0.25	0.33
13	1.99	1.58	0.52	0.34	0.35
14	1.52	1.50	0.43	0.29	0.35
	**DArT**				
1	1.65	1.55	0.40	0.27	0.41
2	1.77	1.58	0.44	0.29	0.38
3	1.62	1.53	0.37	0.25	0.38
4	1.37	1.37	0.25	0.18	0.37
5	2.77	1.69	0.62	0.37	0.38
6	2.56	1.65	0.59	0.35	0.36
7	1.42	1.42	0.29	0.21	0.42
8	2.41	1.60	0.55	0.33	0.35
9	1.29	1.29	0.20	0.14	0.29
10	2.77	1.71	0.64	0.37	0.38
11	1.15	1.10	0.09	0.06	0.08
12	1.84	1.64	0.47	0.30	0.41
13	2.93	1.69	0.64	0.37	0.37
14	1.96	1.61	0.50	0.32	0.38
